# Supplementary material for: Measuring Self-Efficacy for Exercise among Older Adults: Psychometric Properties and Measurement Invariance of a Brief Version of the Self-Efficacy for Exercise (SEE) Scale
Source: Healthcare (Basel). 2024 Aug 17;12(16):1642. doi: 10.3390/healthcare12161642 (PMC11353483; doi:10.3390/healthcare12161642)
Supplement: Supplementary file 1 [file healthcare-12-01642-s001.zip › Table S1.pdf]

Supplemental Materials

Table S1. Descriptive statistics of the SVDS, GSLTPAQ, BFI-2-XS, and the SF-12 and their corresponding items

			Mean	SD	Skewness	Kurtosis
SEE	Self-Efficacy for Exercise		4.99	2.84	0.063	-0.917
		Item 1: Feeling pain when exercising	4.68	3.40	0.216	-1.221
		Item 2: Did not enjoy the exercise	5.47	3.45	-0.076	-1.211
		Item 3: Too busy for exercise	4.60	3.37	0.239	-1.095
		Item 4: Feeling tired	4.62	3.38	0.231	-1.328
		Item 5: Bad mood	5.60	5.60	-0.105	-1.328
GSLTPAQ[a]	LSI		35.66	34.09	1.866	5.568
BFI-2-XS	Open-Mindedness		3.66	0.96	-0.583	-0.221
		Item 5: Aesthetic Sensitivity	3.74	1.33	-0.776	-0.632
		Item 10: Intellectual Curiosity	3.48	1.37	-0.404	-1.117
		Item 15: Creative Imagination	3.77	1.074	-0.710	-0.124
	Conscientiousness		4.03	0.88	-0.688	-0.414
		Item 3: Organization	3.94	1.28	-0.922	-0.467
		Item 8: Productiveness	3.66	1.32	-0.496	-1.111
		Item 13: Responsibility	4.48	0.73	-1.591	2.948
	Extraversion		3.33	0.96	-0.197	-0.653

		Item 1: Sociability	3.04	1.47	0.017	-1.440
		Item 6: Assertiveness	3.44	1.30	-0.435	-0.928
		Item 11: Energy Level	3.49	1.23	-0.440	-0.863
	Agreeableness		3.87	0.75	-0.440	-0.261
		Item 2: Compassion	4.32	0.90	-1.486	2.080
		Item 7: Respectfulness	3.60	1.40	-0.481	-1.232
		Item 12: Trust	3.69	1.11	-0.577	-0.455
	Negative emotionality		2.48	0.99	0.326	-0.626
		Item 4: Anxiety	3.27	1.51	-0.302	-1.394
		Item 9: Depression	2.12	1.32	0.800	-0.759
		Item 14: Emotional Volatility	2.05	1.09	0.966	0.234
SF-12[b]	PCS12		48.60	9.76	-0.848	-0.333
	MCS12		43.41	9.52	-0.479	-0.530

Note. The range of questionnaires' score are respectively: from 0 to 10 for the SEE and from 0 to 5 for the BFI-2-XS. [a]Interpretation of the LSI score: LSI < 14, insufficiently active/sedentary; LSI = 14-23, moderately active; LSI ≥ 24, active. [b]SF-12 raw scores were transformed into T scores (M = 50 and ds = 10) using Ware et al. (1) algorithm.

References

1. Ware, J.E.; Kosinski, M.; Keller, S.D. A 12-Item Short-Form Health Survey: Construction of Scales and Preliminary Tests of Reliability and Validity. *Med Care* **1996**, *34*, 220.
